# Stress pre-conditioning with temperature, UV and gamma radiation induces tolerance against phosphine toxicity

**DOI:** 10.1371/journal.pone.0195349

**Published:** 2018-04-19

**Authors:** Saad M. Alzahrani, Paul R. Ebert

**Affiliations:** 1 The University of Queensland, School of Biological Sciences, St Lucia, QLD, Australia; 2 King Abdulaziz City for Science and Technology (KACST), Nuclear Science Research Institute (NSRI), Riyadh, Saudi Arabia; 3 Plant Biosecurity Cooperative Research Centre, Bruce, ACT, Australia; Washington State University, UNITED STATES

## Abstract

Phosphine is the only general use fumigant for the protection of stored grain, though its long-term utility is threatened by the emergence of highly phosphine-resistant pests. Given this precarious situation, it is essential to identify factors, such as stress preconditioning, that interfere with the efficacy of phosphine fumigation. We used *Caenorhabditis elegans* as a model organism to test the effect of pre-exposure to heat and cold shock, UV and gamma irradiation on phosphine potency. Heat shock significantly increased tolerance to phosphine by 3-fold in wild-type nematodes, a process that was dependent on the master regulator of the heat shock response, HSF-1. Heat shock did not, however, increase the resistance of a strain carrying the phosphine resistance mutation, *dld-1*(*wr4*), and cold shock did not alter the response to phosphine of either strain. Pretreatment with the LD_50_ of UV (18 J cm^-2^) did not alter phosphine tolerance in wild-type nematodes, but the LD_50_ (33 J cm^-2^) of the phosphine resistant strain (*dld-1*(*wr4*)) doubled the level of resistance. In addition, exposure to a mild dose of gamma radiation (200 Gy) elevated the phosphine tolerance by ~2-fold in both strains.

## Introduction

Phosphine (PH_3_) gas is an ideal fumigant for the control of insect pests of stored commodities due to the low cost of application, ease of use and the lack of chemical residues. Phosphine is also environmentally benign as it decomposes to phosphate. These properties are not matched by any other potential fumigant, leading to heavy dependence on phosphine fumigation around the world [1]. Phosphine is a metabolic poison that affects cellular respiration [2, 3]. It may also disrupt neural acetylcholine signaling [4] or cause damage to DNA [5]. In addition, phosphine is known to cause oxidative damage [4]. The diversity of potential mechanisms makes it difficult to predict interactions between phosphine and other treatments.

We have chosen to investigate the effect of pre-exposure to diverse stressors for two purposes: firstly, to increase our understanding of the toxic mechanism of phosphine, and secondly, to identify positive interactions that might be useful in practice to improve the efficacy of phosphine. In this work, we use the free-living nematode *Caenorhabditis elegans* as a model organism to investigate the effect of pretreatment with heat, cold, UV and gamma radiation on phosphine sensitivity. Each of these treatments has been used commercially to protect stored commodities except UV radiation. High temperature is used to control pest infestation whereas cooling of grain in warm climates is typically used to suppress growth and reproduction of pest insects to slow infestation [6]. Gamma irradiation is used on a limited scale as a quarantine treatment for stored grain [7–9].

These stressors may interact with phosphine toxicity and genetic resistance to phosphine in a variety of ways. These include hormesis, a phenomenon where a living organism acquires tolerance to a stressor following challenge with a sublethal dose/concentration of the same or a different stressor [10]. In addition, synergism, cross-resistance and sensitization to phosphine have each been observed under various conditions that are discussed below.

The response to heat stress has been more exhaustively studied and in a wider range of species than has any other stress response. Pretreatment with heat increases the thermotolerance of *C*. *elegans* and results in an extended lifespan [10]. Heat shock, where organisms are expose to lethal high temperature for a short nonlethal period, also enhances the resistance to a variety of chemical and physical stressors [11]. Pre-exposure to cold stress can also induce tolerance to subsequent stress in the exposed organism [12]. The protective response that is induced by pre-exposure to extreme temperature is mediated by the production of heat shock proteins (*HSP*s). These proteins protect cells from not only extreme temperature but also other stressors [11, 13, 14].

Exposure of *C*. *elegans* to ultraviolet radiation at low doses inhibits their fertility and at high doses is lethal [15]. When wild-type *C*. *elegans* is exposed to 40 J m^-2^ of UV light, the survival rate is 2.4% [16]. The nematodes that do survive exhibit an increase in lifespan, indicating that exposure to UV light has triggered a protective defense mechanism. A mild dose of UV also induces a protective response in *C*. *elegans* against oxidative damage caused by exposure to heavy metals [17]. Furthermore, exposure of *C*. *elegans* to UV radiation early in development inhibits aerobic respiration throughout development as determined by decreased oxygen consumption [18].

Cross-resistance between phosphine and gamma radiation has been observed in a phosphine-resistant strain of the lesser grain borer relative to its susceptible counterpart. The resistant strain was able to withstand the DNA damage caused by gamma irradiation as assessed by single-cell electrophoresis (comet assay). Furthermore, cross-adaptation of *Drosophila* sp. to heat and oxidative stress was observed after pretreatment with gamma radiation. The flies became more tolerant of oxidative stress induced by superoxide radical (O^-^_2_) after exposing to gamma rays at an early life stage [14].

In this paper, experiments were designed to identify major stress response pathways that interact with the response to phosphine exposure in either phosphine susceptible or resistant animals. The approach was to subject *C*. *elegans* to a shock of lethal magnitude, but for a sublethal period. The response to a subsequent exposure to phosphine was then monitored relative to the unshocked control to identify preconditioning effects. We test high and low temperature stress as well as exposure to UV and gamma radiation in both a wild-type and a phosphine-resistant strain. We find that preconditioning due to heat stress is mediated through heat shock response factors.

## Materials and methods

### *C*. *elegans* strains and culture conditions

The strains of *C*. *elegans* used in this study are the phosphine susceptible wild-type strain N_2_ and the phosphine-resistant strain *dld-1*(*wr4*) [2]. Also, three strains with a genetically modified heat shock response, The three strains, (RB791 (*hsp-16*.*48*), RB1104 (*hsp-3*) and PS3551 (*hsf-1*)), were provided by the *C*. *elegans* Genetic center (CGC), which is funded by the National Institutes of Health (NIH) Office of Research Infrastructure Programs (P40 OD010440). Before phosphine treatment, Synchronized L_1_ worms were prepared as previously described in [19]. The L_1_ nematodes were cultured on 6 cm NGM agar plates (0.3% NaCl, 0.25% peptone, 5 mg/ml cholesterol, 1 mM CaCl_2_, 1 mM MgSO_4_, 1.7% agar) seeded with *E*. *coli* strain OP50 and grown at 20°C for 24 hours (L_2_ stage) or 48 hours (L_4_ stage).

### Chemical

The phosphine gas used in these experiments was generated from aluminum phosphide tablets (570g/kg aluminum phosphide, BEQUISA Co. (GASTION), Brazil). A fragment of aluminum phosphide tablet was placed in one liter of 5% sulfuric acid in a Valmas chamber [20]. The gas was collected in an air-tight receptacle sealed with silicon septum that allowed gas to be withdrawn with a syringe.

### Pretreatments

#### Heat and cold shock

Prior to phosphine fumigation, developmentally synchronized L_4_ stage *C*. *elegans* were incubated on NGM agar for 4 hours at30°C. In the case of cold shock, the nematodes were maintained in an incubator at 10±0.5°C for 4 hours. The stressed nematodes were then moved to the normal temperature of 20°C for 4 hours.

#### Ultraviolet radiation

UV irradiation was carried out as described by Wang et. al. [17]. Synchronized L_2_ stage nematodes of wild-type (N2) and phosphine-resistant (*dld-1*(*wr4*)) strains were irradiated with 18 and 33 J cm^-2^ UV light respectively (XLE-Series UV crosslinker, Spectronics Co.). These doses represent the LD_50_ for each strain S1 Fig, S3 Table). After irradiation, the worms were allowed to recover at 20°C for 24 hours at which point they had reached the L_4_ stage and were ready for phosphine fumigation as described below.

#### Gamma radiation

L_1_ stage nematodes of wild-type (N2) and phosphine-resistant (*dld-1*(*wr4*)) strains were irradiated with gamma rays as described in [10]. The gamma dosage was 200 Gy utilizing a cobalt-60 Gammacell-220 irradiator (Atomic Energy of Canada Ltd.). Subsequent to gamma irradiation, the nematodes were incubated at 20°C for 48 hours to reach the L_4_ stage for the phosphine fumigation.

### Phosphine fumigation

Phosphine exposure was carried out as previously described in [20]. The plates were placed in air-tight desiccators into which a measured amount of phosphine gas was injected. In all cases, the volume of gas that was injected into the chamber was less than 0.2% of the volume of the chamber. The concentrations of gas that were used were 0, 50, 100, 200, 400, 600, 800, 1000, 1200,1600, 2000, 2500, 3200 and 6400 ppm. Fumigations were carried out for 24 hours, in line with established resistance monitoring protocols in pest insects. Following the fumigation, the nematodes were transferred to fresh air to recover for 48 hours.

### Statistical analysis

Each experiment was repeated three times, and each trial contained two replicates per strain per phosphine concentration. After the forty-eight hour recovery period, Automated WormScan was utilized for mortality scoring as described in [21, 22]. Briefly, the treated worms in the six centimeters plates were scanned and the individuals that did not respond to the light stimulus in the ten minutes period between scans were scored as dead. To determine the median lethal concentrations (LC_50_) of phosphine, probit analysis was carried out on exposures that resulted in 0.1% to 99.9% average mortality [23]. The analysis was carried out using the software LdP Line (copyright 2000 by Ehab Mostafa Bakr, Cairo, Egypt, http://www.ehabsoft.com/ldpline/), and graphs were generated using the software SigmaPlot [24]. The effects of pretreatments on the LC_50_ of phosphine were computed by one-way ANOVA followed by Dunnett’s multiple comparisons. An unpaired t-test was performed to compare the heat shock effect on phosphine resistance in each of the heat shock mutants. These comparative tests was performed using GraphPad Prism (version 7.03 for Windows, GraphPad Software, La Jolla California USA, www.graphpad.com).

## Results

### Heat shock

**Fig 1:**
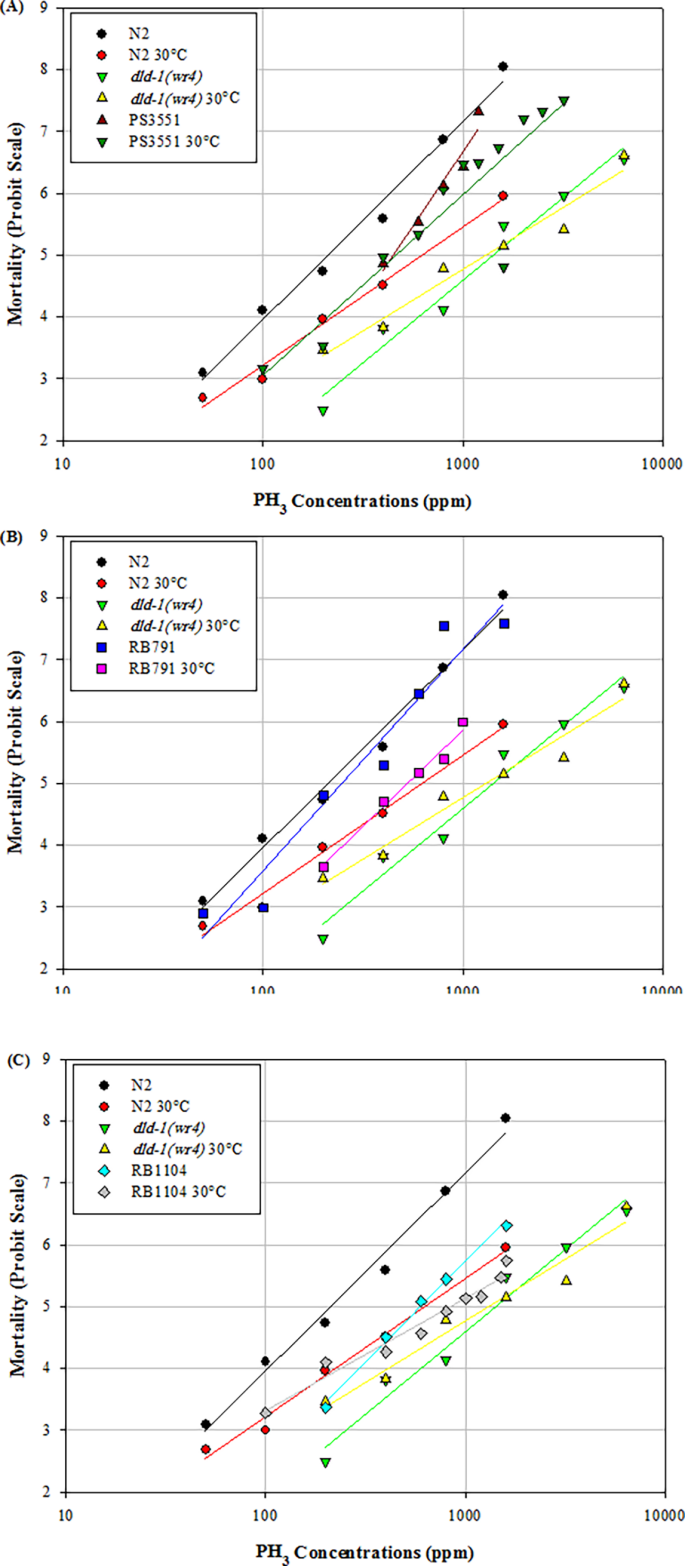
Heat shock preconditioning against phosphine-induced mortality of *C*. *elegans* in wild-type, phosphine-resistant and heat shock response mutants. A four-hour heat shock at 30^◦^C was followed by a 4 hour recovery period, after which the nematodes were subjected to 24 hour exposure to phosphine. Mortality was scored after a further 48 hour recovery period, either without or with heat shock preconditioning. Wild-type (N2), phosphine-resistant (*dld-1(wr4)*). (A) PS3551 (*hsf-1*) (B) RB791 (*hsp-16*.*48*) (C) RB1104 (*hsp-3*).

**Fig 2:**
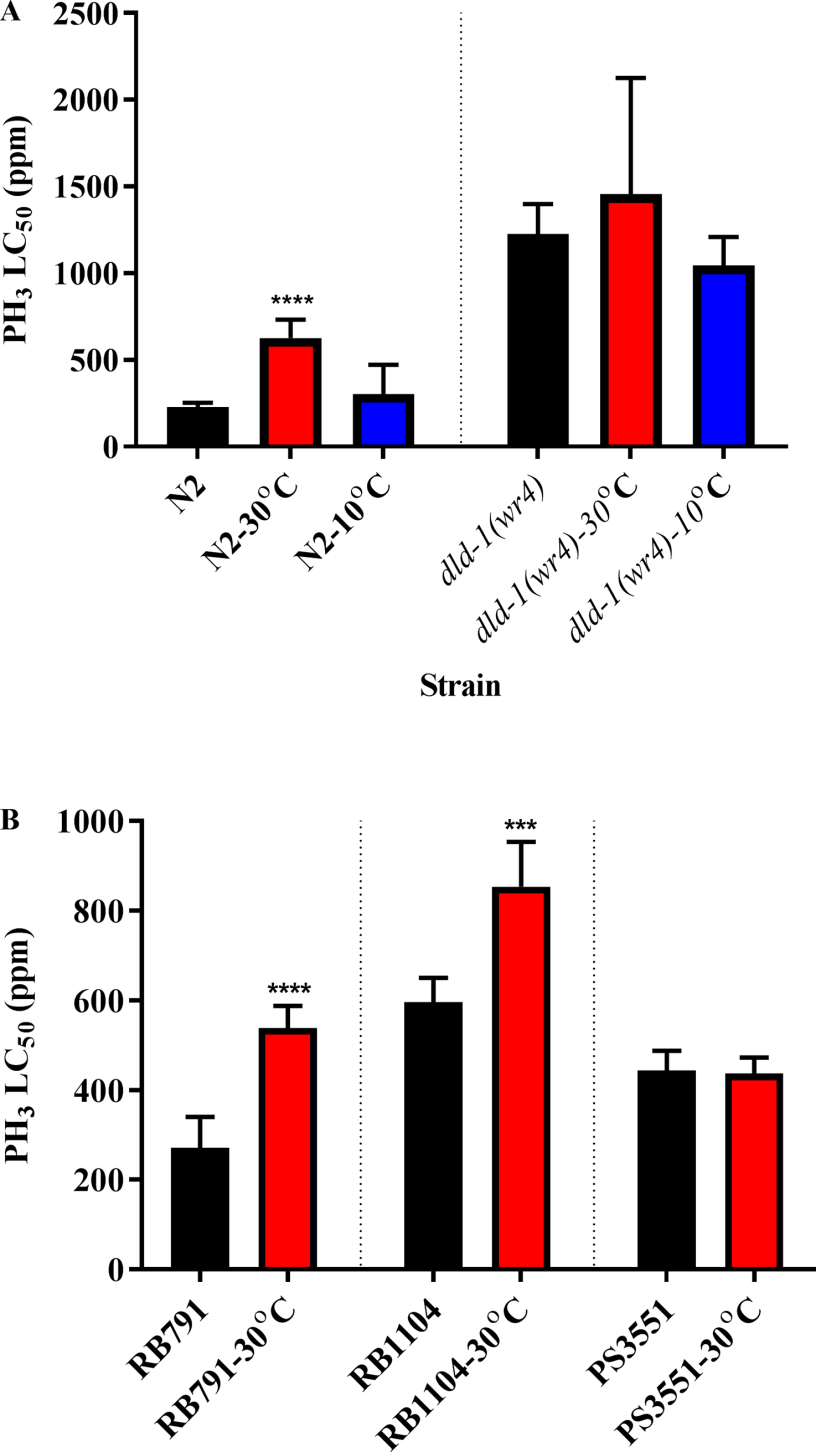
Analysis of LC_50_ value for heat shock preconditioning against phosphine-induced mortality of *C*. *elegans*. (A) Comparison of the wild-type and the phosphine-resistant mutant and (B) heat shock mutants either without or with heat or cold preconditioning. A four-hour heat shock at 30^◦^C was followed by a 4 hour recovery period, after which the nematodes were subjected to phosphine exposure for 24 hours. Mortality was scored after a further 48 hour recovery period. The LC_50_ value for each strain is shown, either without or with heat shock preconditioning. Error bars represent the 95% confidence intervals for each LC_50_ data point. One-way ANOVA followed by Dunnett’s multiple comparisons for the LC_50_ values, ****p < 0.0001. Wild-type (N2), phosphine-resistant (*dld-1(wr4)*). Unpaired t-test was used to compare the LC_50_ values of each heat shock mutant strain, ****p < 0.0001, ***p < 0.001. RB791 (*hsp-16*.*48*), RB1104 (*hsp-3*) and PS3551 (*hsf-1*).

Whereas the wild-type animals showed strong heat shock preconditioning, the phosphine-resistant *dld-1(wr4)* mutant exhibited a mild increase in resistance that was not statistically significant. Without heat shock, the wild-type animals showed a normal concentration-dependent response to phosphine exposure—an LC_50_ of 229 ppm after fumigation for 24 hours at 20°C. On the other hand, if wild-type animals were given a 4-hour heat shock at 30°C, then allowed to recover for four hours prior to fumigation, the LC_50_ increased to 625 ppm phosphine. The 2.7 fold induced tolerance was statistically significant at (*P* = 0.05) (Figs 1 and 2A, S1 Table). In contrast, exposing the phosphine-resistant strain to the same 30°C heat shock before fumigation resulted in a statistically insignificant increase in the LC_50_ from 1227 ppm in the absence of heat shock to 1456 ppm when heat shock was applied (Figs 1 and 2A, S1 Table). The slope of the response curve of the wild-type strain decreased following heat shock, indicating an increase in phenotypic diversity within the population. The result was a slope that was similar to that of the phosphine response curves of the resistant strain, either with or without heat shock. These slopes differed markedly from the response of the wild-type strain to phosphine in the absence of heat shock (Fig 1, S1 Table).

We then tested the effect of mutations in two heat shock response effector genes on the heat shock-induced tolerance toward phosphine. As with the wild-type strain, the *hsp-1*6.*48* mutant strain (RB791) showed increased tolerance to phosphine after heat shock. The LC_50_ of this strain toward phosphine in the absence of heat preconditioning was 271 ppm, whereas after exposure to a 30°C shock the LC_50_ almost doubled to 539 ppm (Fig 1B, S1 Table). Similarly, the *hsp-3* strain (RB1104) showed induced resistance due to heat preconditioning, but the magnitude of the response was only 1.4-fold, from an LC_50_ of 596 ppm to 854 ppm phosphine (Fig 1C, S1 Table). The slope of the response curve, once again, was shallower when individuals of this strain were exposed to heat shock preconditioning. On the contrary, elimination of the master regulator of the heat shock response, HSF-1, completely eliminates the heat shock preconditioning effect. Thus, the LC_50_ of the *hsf-1* mutant strain (PS3551) was 437 ppm phosphine in the absence of heat shock at 30°C and 444 ppm phosphine following heat shock, which are statistically indistinguishable (Fig 1A, S1 Table).

### Cold shock

**Fig 3:**
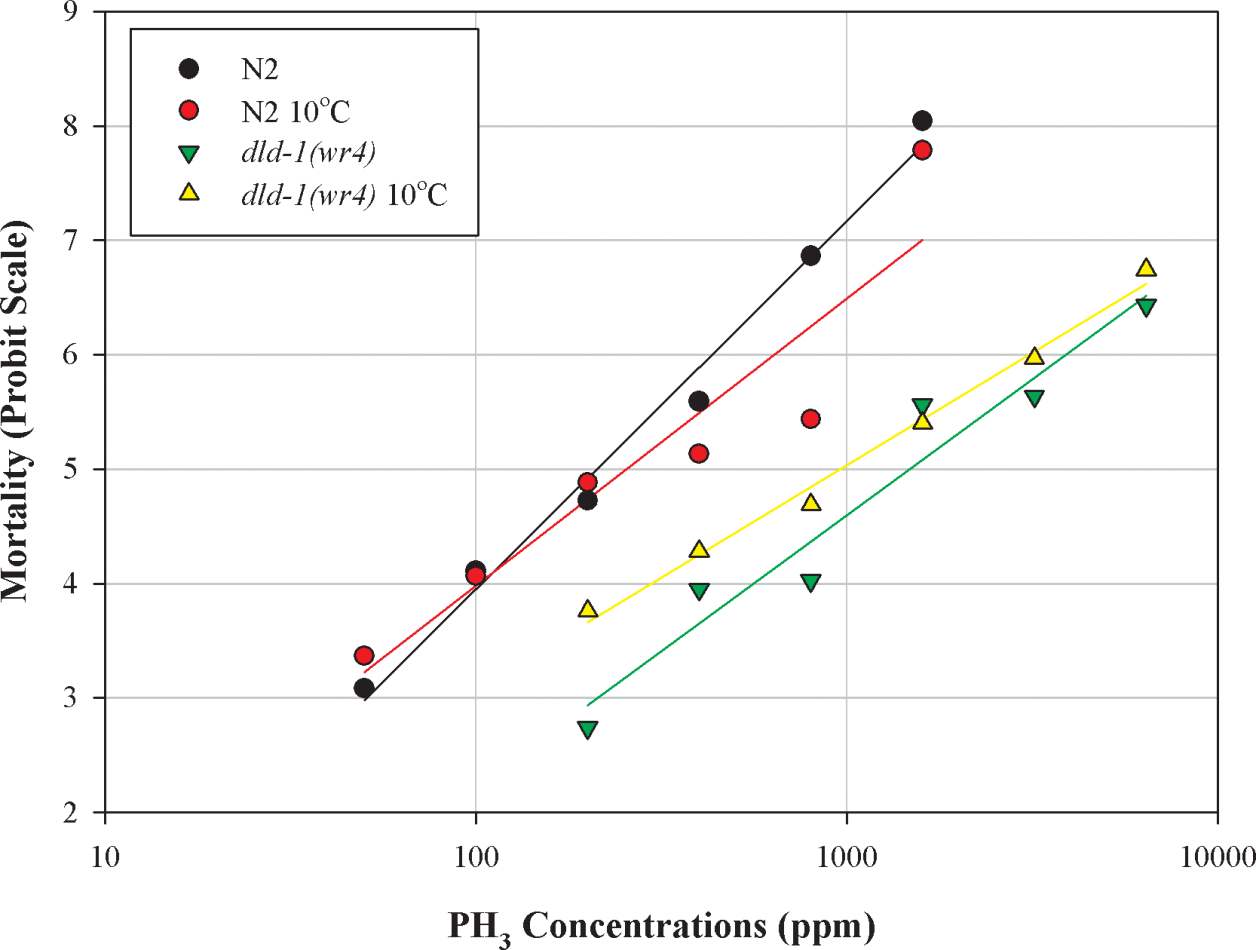
Effect of cold shock on phosphine-induced mortality of wild-type *C*. *elegans* and the phosphine-resistant *dld-1* mutant. A four-hour cold shock at 10^◦^C was followed by a 4 hour recovery period, after which the nematodes were subjected to 24 hour exposure to phosphine. Mortality was scored after a further 48 hour recovery period. Wild-type (N2), phosphine-resistant (*dld-1(wr4)*).

The pre-treatment of wild-type animals with a 4 hour cold shock at 10°C resulted in no significant increase in the LC_50_ (304 ppm) in response to phosphine relative to the LC_50_ of the untreated control (229 ppm). Unlike heat shock, cold shock of the phosphine-resistant strain may have caused slight sensitization to phosphine, with a decrease in the LC_50_ from 1227 to 1044 ppm, though the effect did not reach statistical significance (Fig 3, S1 Table).

### Ultraviolet radiation

**Fig 4:**
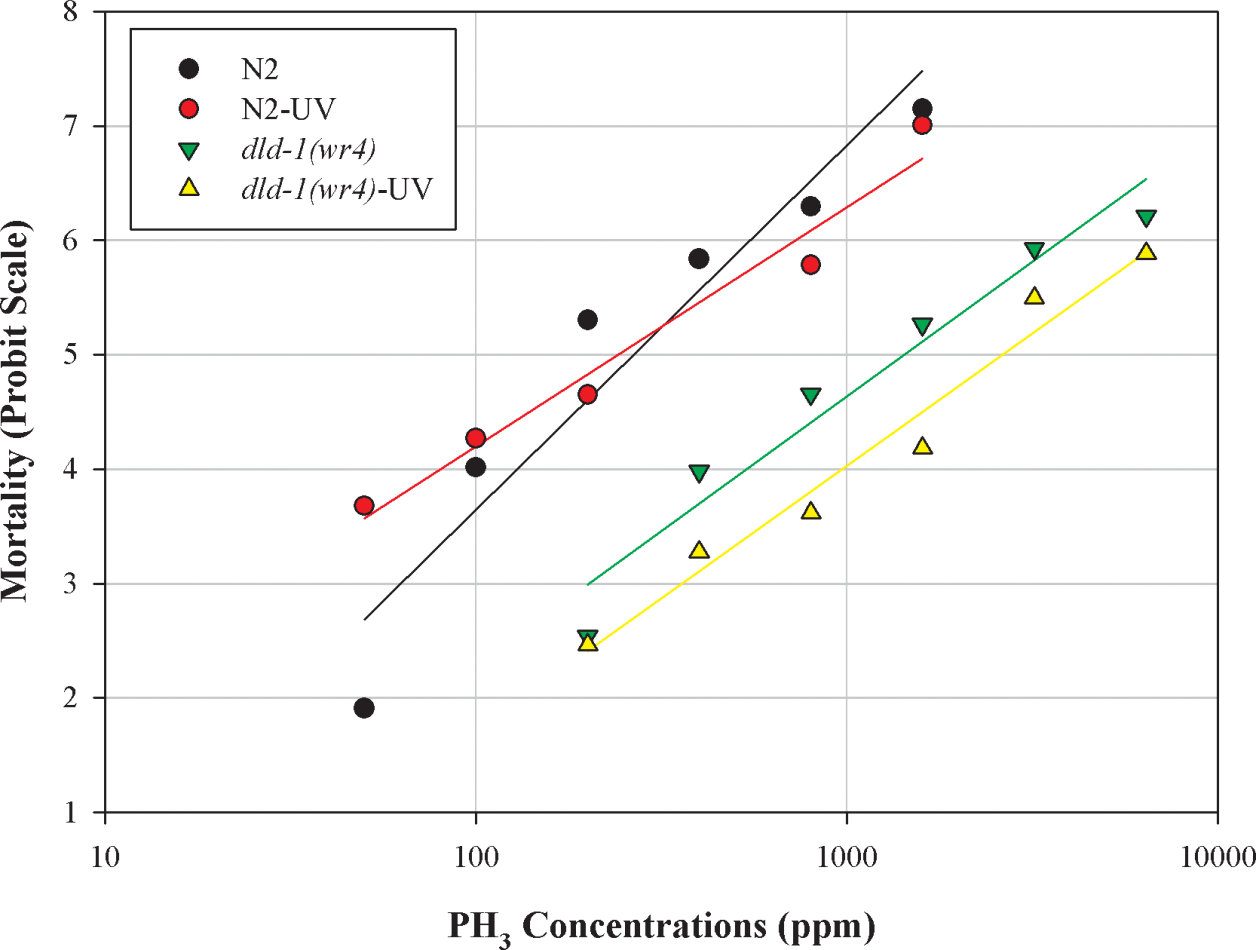
Effect of UV light on phosphine-induced mortality of wild-type *C*. *elegans* and the phosphine-resistant *dld-1* mutant. *C*. *elegans* were exposed to 18 and 33 J cm^-2^ UV radiation 24 hours, after which the nematodes were subjected to 24 hour phosphine exposure. Mortality was scored after a further 48 hour recovery period. Wild-type (N2), phosphine-resistant (*dld-1(wr4)*).

**Fig 5:**
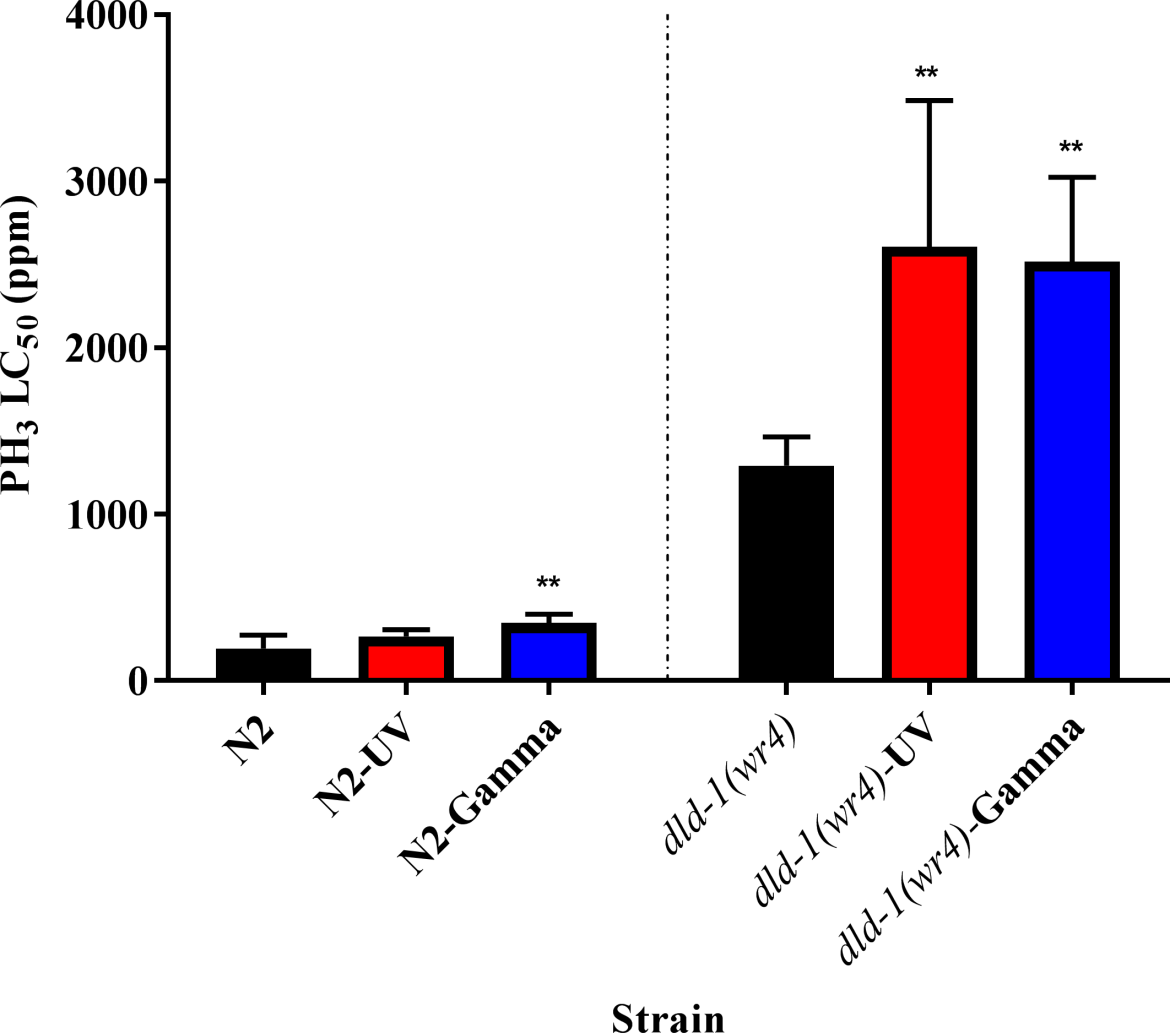
Effect of UV and gamma radiation on phosphine-induced mortality of C. elegans in wild-type and the phosphine-resistant *dld-1* mutant. Nematodes were exposed to a range of dosages of either UV light or gamma radiation at the L_1_ stage. Mortality was assessed 48hrs after exposure as lack of movement in response to a bright light stimulus. LC_50_ values are shown and the error bars represent the 95% confidence intervals for each LC_50_ data point. Wild-type (N2), phosphine-resistant (*dld-1(wr4)*). One-way ANOVA followed by Dunnett’s multiple comparison test for the LC_50_ values, **p < 0.01.

The response to UV radiation manifests over an extended period. To determine whether exposure to UV light results in induced preconditioning to phosphine. L_2_ stage nematodes, wild-type, and mutant were given a burst of UV radiation at their respective LD_50_ values, 18 and 33 J cm^-2^ (S1 Fig, S3 Table), after which they were allowed to grow under standard conditions to the L_4_ stage [19]. UV pretreatment did not affect the response to phosphine of the wild-type strain. The LC_50_ for the wild-type control was 195 ppm, which increased to 266 ppm following UV pre-treatment, but the difference was not statistically significant. On the contrary, the phosphine-resistant strain showed preconditioning against phosphine toxicity in response to UV exposure. The LC_50_ with UV pretreatment significantly increased to 2607 ppm from 1291 ppm without UV pre-treatment (Figs 4 and 5, S2 Table).

### Gamma radiation

**Fig 6:**
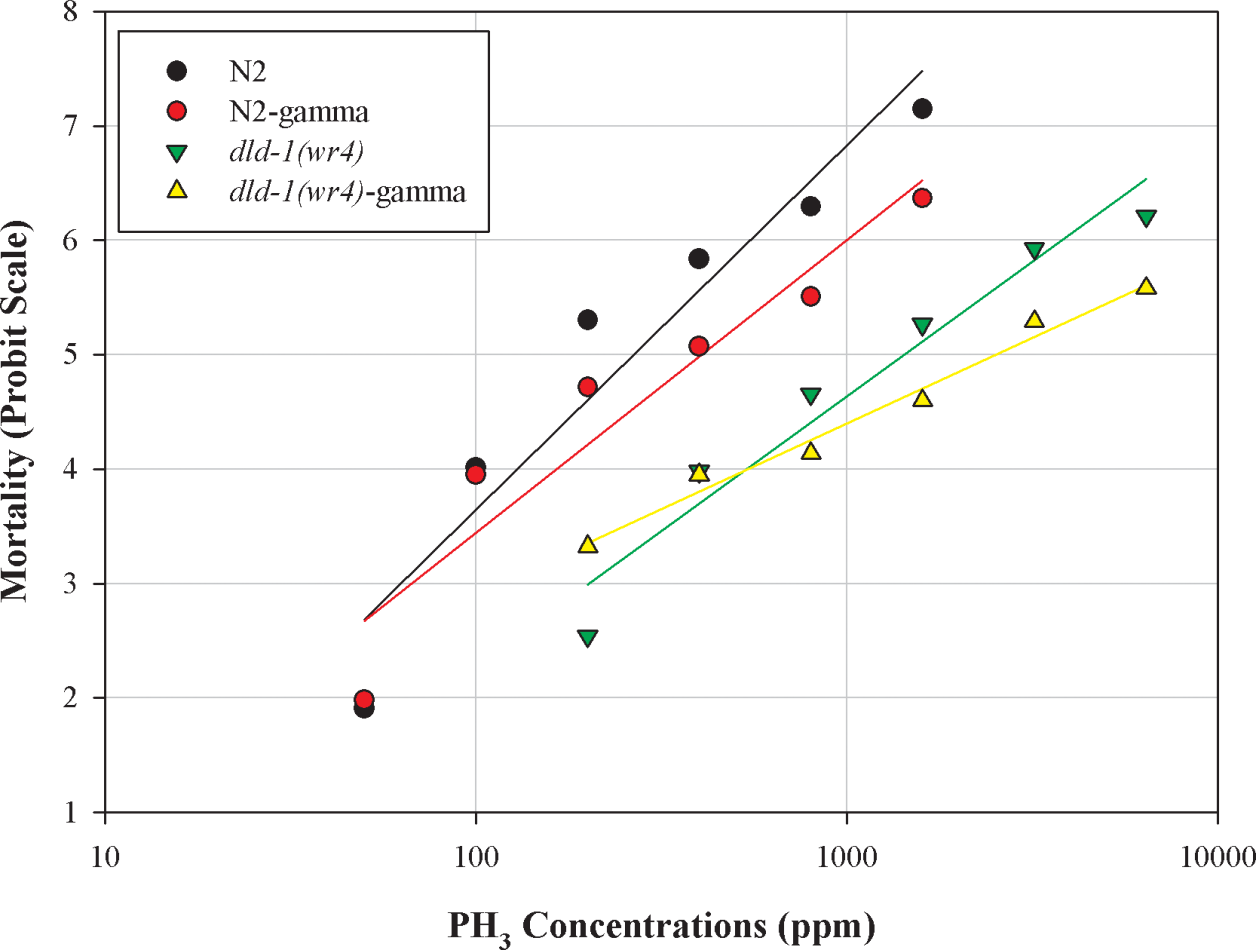
Exposure to gamma radiation induces tolerance to phosphine exposure in the wild-type and the phosphine-resistant worms. *C*. *elegans* were exposed to 200 Gy gamma radiation 24 hours, after which the nematodes were subjected to 24 hour exposure to phosphine. Mortality was scored after a further 48 hour recovery period. Wild-type (N2), phosphine-resistant (*dld-1(wr4)*).

Wild-type nematodes exposed to a pretreatment of 200 Gy of gamma radiation show an increase in the LC_50_ of about 1.4-fold compared to nematodes that have not been pretreated with gamma radiation. The LC_50_ rose from 195 to 346 ppm in response to pretreatment, which was statistically significant. In the phosphine-resistant strain, the same effect was observed with a slightly greater magnitude. Gamma radiation pretreatment increased the LC_50_ to phosphine 2-fold compared to nematodes that had not been pretreated, with an increase in LC_50_ from 1291 to 2518 ppm. Once again, the observed difference was statistically significant (Figs 5 and 6, S2 Table). In both strains, the slopes of the response curves became significantly shallower in response to gamma radiation pretreatment.

## Discussion

The adaptive response of organisms to stress has been widely studied, including communication between and cross-induction of stress response pathways [10, 13, 14, 17]. In this research, we tested the ability of four distinct stresses to induce cross-resistance against the agriculturally important fumigant phosphine, with the goal of gaining a deeper understanding of how resistance is mediated. We monitored the effect of preconditioning against the toxicity of phosphine in a wild-type strain of *C*. *elegans* (N2), as well as in a phosphine-resistant mutant (*dld-1(wr4)*). The orthologue of the *dld-1* gene is also a major phosphine resistance factor in pest insects of stored grain, making these studies of stress-induced tolerance of practical importance to pest control.

Whereas exposure to cold did not alter the response to phosphine, we found that pretreatment with heat, UV light and gamma radiation each altered the response of the nematodes to subsequent exposure to phosphine gas, but in unique ways. We chose heat shock-induced preconditioning for further study because it has been exhaustively investigated and because heat stress is a condition likely to be encountered by pest insects in the field. We initially screened seven strains that had been mutated in various heat shock response genes to identify genes important to heat shock preconditioning against phosphine toxicity. Three mutants were selected for further study, as heat preconditioning influenced their responses to phosphine, each in a unique way. Two of the strains carried a mutation in one of the heat shock response effector genes, whereas the third had a mutation that disrupted HSF-1, the master regulator of the heat shock response [25] S4 Table.

When organisms are exposed to heat shock, their cells produce heat stress defense proteins as an adaptive mechanism [26]. The expressed proteins provide not only protection against heat stress, but also to a range of other stressors as well. Thus, heat shock is known to induce cross-tolerance to secondary abiotic stresses [11]. We observe that heat pretreatment made wild-type nematodes more resistant to phosphine by 2.7-fold. The phosphine-resistant strain, however, did not exhibit heat shock induced preconditioning against phosphine. This result suggests that the heat shock inducible defenses that lead to phosphine resistance are constitutively upregulated in the strain carrying the phosphine-resistance mutation *dld-1*(*wr4*) that was used in this study. The fact that strongly phosphine resistant pest insects also carry mutations in the *dld-1* orthologue suggests that heat stress may induce resistance in susceptible insects, but is unlikely to exacerbate the resistance phenotype in insects that already exhibit strong resistance.

When an organism is exposed to heat stress, the endoplasmic reticulum (ER) environment is disturbed, an event that can result in interruption of the protein folding process. The accumulation of unfolded proteins in the ER triggers the unfolded protein response (UPR). One of the primary processes of the UPR is the upregulation of chaperones that will bind to the unfolded proteins and prevent their transport [27]. *HSP* genes are transcriptionally upregulated in response to ER stress [28]. There is also a distinct UPR in the mitochondria that includes a unique set of chaperones that protect against protein unfolding in that cellular compartment [29].

We included in this study, three strains each of which contained a mutation in one of three genes, *hsp-16*.*48*, *hsp-3* and *hsf-1*. The HSP-16 protein is a member of the α-crystallin family of small heat shock proteins (sHSPs). These proteins are strongly induced in *C*. *elegans* in response to heat stress and contribute to stress resistance and longevity [30, 31]. One of the effects of phosphine poisoning is necrosis of tissues in the exposed organism [32]. Kourtis et al. [31] concluded in their study that a single sHSP is sufficient to protect against necrotic insults, it may be that phosphine-induced necrosis is prevented by upregulation of chaperones, thereby increasing tolerance toward phosphine.

We have demonstrated a clear relationship between the heat shock response and the induction of resistance to phosphine. Heat shock is able to induce phosphine resistance in wild type nematodes, but only in the presence of HSF-1. This supports the notion that the induced resistance to phosphine occurs through the heat shock response system. Heat shock is, however, unable to further increase the resistance level of the phosphine resistant strain. It is interesting to note that reactive oxygen species (ROS) are the mediators of phosphine toxicity [4]. ROS can also induce the heat shock response and the response itself protects against the damaging effects of ROS. In the wild type animals, it seems that induction of anti-ROS defenses provides a significant level of resistance against phosphine exposure. Our results also indicate that the heat shock response is either constitutively activated in the resistant strain or that an alternative anti-ROS defense is used that makes induction the heat shock response redundant. An alternative explanation is that phosphine does not induce ROS generation in the mutant, which would simply make the heat shock inducible anti-ROS defense system unnecessary [33, 34].

In the case of cold shock, we observed no significant difference in phosphine sensitivity. This could be due to the fact that 10°C is within the normal temperature range that *C*. *elegans* experiences in the environment [35], which makes it not stressful enough to trigger a temperature stress defense mechanism.

Pretreatment with ultraviolet radiation has no hormetic effect on phosphine resistance in wild-type nematodes, which is consistent with previous findings [10]. Their proposed explanation is that *C*. *elegans* is a soil-borne organism that is not exposed to damaging amounts of UV radiation in its natural habitat. As a result, there was insufficient selective pressure to drive the evolution of a genetic response to resist UV stress. Others [17], however, have reported that pretreating *C*. *elegans* with UV light increases the resistance of worms to neurotoxic metals and decreases the level of oxidative stress resulting from exposure to these metals. It is important to note that the authors assessed the effect of these neurotoxins on the locomotory behavior, whereas we assessed the effect on mortality due to phosphine exposure. As a result, the two results are not directly comparable.

In this research, pretreatment with gamma radiation produced cross-protection against phosphine in the wild-type strain (1.4-fold) as well as the phosphine-resistant mutants (2-fold). Similar to UV exposure, gamma radiation pretreatment of the phosphine-resistant mutants has doubled their resistance to phosphine. As with heat shock, gamma radiation can trigger living cells to produce heat shock proteins and these proteins are responsible for cross-tolerance to a variety of stressors [14], though our experiments do not rule out alternative explanations.

Controlled storage temperature and gamma radiation are used to disinfest stored products of insect pests. In addition, the insects may encounter temperature extremes and exposure to UV light in the environment. We find that these stresses can significantly affect the efficacy of phosphine fumigation and we identify a stress response pathway through which tolerance to phosphine can be induced. Our findings can contribute to more effective phosphine fumigation by taking into account any planned or unplanned pre-exposure to environmental stresses.

## Supporting information

S1 Fig Dose-dependent mortality of *C*. *elegans* due to exposure to UV light. Nematodes were exposed to a range of UV dosages at the L1 stage. Mortality was assessed 48hrs after UV exposure as lack of movement in response to a bright light stimulus. Wild-type (N2), phosphine-resistant (*dld-1(wr4)*).
(TIF)

S1 Table Phosphine LC_50_ values and resistance factor for *C*. *elegans* strains with and without preconditioning. One way ANOVA followed by Dunnett’s multiple comparison test was used to identify significant differences in LC_50_ values due to phosphine exposure between the wild-type and *dld-1(wr4)* strains, as well as between treated and untreated animals. An unpaired t-test was used to compare the LC_50_s values between pretreated or unpretreated heat shock response mutants, PS3551, RB1104 and RB791.
(DOCX)

S2 Table Phosphine LC_50_ values and resistance factor for *C*. *elegans* strains with and without radiation preconditioning. One-way ANOVA followed by Dunnett’s multiple comparisons to compare the LC_50_ with the pretreatments LC_50_s for the wild-type and *dld-1(wr4)*, ANOVA followed by Dunnett’s multiple comparison test was used to identify significant differences in LC_50_ values due to phosphine exposure between the wild-type and *dld-1(wr4)* strains.
(DOCX)

S3 Table UV induced mortality of wild-type and phosphine-resistant strains of *C*. *elegans*. (DOCX)

S4 Table *C*. *elegans* mutants of the heat shock response were screened for a change in induced tolerance toward phosphine. A screening phosphine-bioassay in *C*. *elegans* mutants, that have been characterized in the *C*. *elegans* Genetic Center with genetic-mutated background in regard to heat shock response. Mutants with unique heat shock response to phosphine toxicity were chosen.
(DOCX)

S1 Dataset Raw data (UV-preconditioning). (XLSX)

S2 Dataset Raw data (gamma-preconditioning). (XLSX)

S3 Dataset Raw data (temperature-preconditioning). (XLSX)
