# Supplementary figures and images for: Stress pre-conditioning with temperature, UV and gamma radiation induces tolerance against phosphine toxicity
Source: PLoS One. 2018 Apr 19;13(4):e0195349. doi: 10.1371/journal.pone.0195349 (PMC5909616; doi:10.1371/journal.pone.0195349)

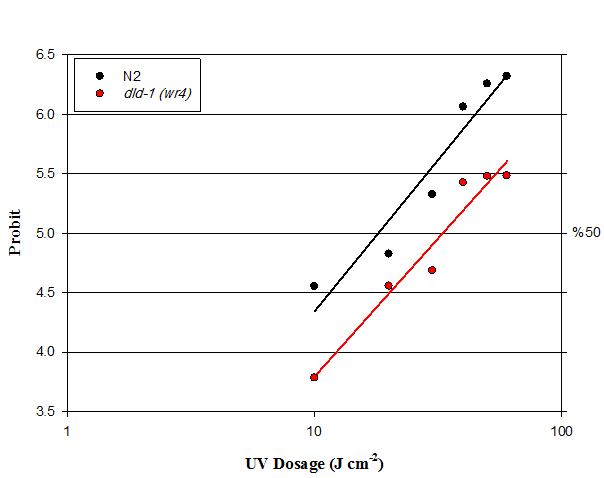

Supplement: S1 Fig — Nematodes were exposed to a range of UV dosages at the L1 stage. Mortality was assessed 48hrs after UV exposure as lack of movement in response to a bright light stimulus. Wild-type (N2), phosphine-resistant (dld-1(wr4)). (TIF) [file pone.0195349.s001.tif]
