# Supplementary material for: Stress pre-conditioning with temperature, UV and gamma radiation induces tolerance against phosphine toxicity
Source: PLoS One. 2018 Apr 19;13(4):e0195349. doi: 10.1371/journal.pone.0195349 (PMC5909616; doi:10.1371/journal.pone.0195349)
Supplement: S1 Table — One way ANOVA followed by Dunnett’s multiple comparison test was used to identify significant differences in LC50 values due to phosphine exposure between the wild-type and dld-1(wr4) strains, as well as between treated and untreated animals. An unpaired t-test was used to compare the LC50s values between pretreated or unpretreated heat shock response mutants, PS3551, RB1104 and RB791. (DOCX) [file pone.0195349.s002.docx]

Supporting information

**S1 Fig:** **Dose-dependent mortality of *C. elegans* due to exposure to UV light.** Nematodes were exposed to a range of UV dosages at the L1 stage. Mortality was assessed 48hrs after UV exposure as lack of movement in response to a bright light stimulus. Wild-type (N2), phosphine-resistant (*dld-1(wr4)*).

S1 Table: Phosphine LC_50_ values and resistance factor for *C. elegans* strains with and without preconditioning. One way ANOVA followed by Dunnett’s multiple comparison test was used to identify significant differences in LC_50_ values due to phosphine exposure between the wild-type and *dld-1(wr4)* strains, as well as between treated and untreated animals. An unpaired t-test was used to compare the LC_50_s values between pretreated or unpretreated heat shock response mutants, PS3551, RB1104 and RB791.

| Strain | Pre-treatment temperature (°C) | LC_50_ Phosphine  (95% confidence interval) (ppm) | Slope±SE | X^2^ | R | RF^1^ |
| --- | --- | --- | --- | --- | --- | --- |
| Wild-type (N2) | Not pre-treated | 229 (206-255) | 2.88±0.17 | 3.12 | 0.99 |  |
|  | 30 | 625 (534-749)^****^ | 2.28±0.18 | 1.24 | 0.99 | 2.7 |
|  | 10 | 304 (181-518) | 1.96±0.13 | 22.47 | 0.93 | 1. 3 |
| Phosphine-resistant  *(dld-1(wr4))* | Not pre-treated | 1227 (1064-1409) | 2.47±0.17 | 5.35 | 0.99 |  |
|  | 30 | 1456 (927-2266) | 1.96±0.14 | 7.89 | 0.98 | 1.2 |
|  | 10 | 1044 (892-1221) | 1.75±0.14 | 2.44 | 0.99 | 0.85 |
| PS3551 (*hsf-1*) | Not pre-treated | 444 (397-485) | 4.42±0.38 | 1.82 | 0.98 |  |
|  | 30 | 437 (401-473) | 3.41±0.19 | 11.68 | 0.99 |  |
| RB1104 (*hsp-3*) | Not pre-treated | 596 (544-653) | 3.29±0.27 | 1.48 | 0.99 |  |
|  | 30 | 854 (763-962)^***^ | 1.83±0.14 | 9.42 | 0.98 | 1.4 |
| RB791  (*hsp-16.48*) | Not pre-treated | 271 (206-344) | 3.78±0.24 | 22.48 | 0.97 |  |
|  | 30 | 539 (492-589) ^****^ | 3.17±0.29 | 3.16 | 0.99 | 2 |

^1^Resistance factor to the unpretreated worms for each strain (LC_50_ for pre-treated/LC_50_ unpretreated).

**^***^*p* < 0.001, ^****^*p* < 0.0001, ppm parts per million**
