# Supplementary material for: Stress pre-conditioning with temperature, UV and gamma radiation induces tolerance against phosphine toxicity
Source: PLoS One. 2018 Apr 19;13(4):e0195349. doi: 10.1371/journal.pone.0195349 (PMC5909616; doi:10.1371/journal.pone.0195349)
Supplement: S2 Table — One-way ANOVA followed by Dunnett’s multiple comparisons to compare the LC50 with the pretreatments LC50s for the wild-type and dld-1(wr4), ANOVA followed by Dunnett’s multiple comparison test was used to identify significant differences in LC50 values due to phosphine exposure between the wild-type and dld-1(wr4) strains. (DOCX) [file pone.0195349.s003.docx]

S2 Table: Phosphine LC_50_ values and resistance factor for *C. elegans* strains with and without radiation preconditioning. One-way ANOVA followed by Dunnett’s multiple comparisons to compare the LC_50_ with the pretreatments LC_50_s for the wild-type and *dld-1(wr4)*, ANOVA followed by Dunnett’s multiple comparison test was used to identify significant differences in LC_50_ values due to phosphine exposure between the wild-type and *dld-1(wr4)* strains.

| Strain | Pre-treatment | Phosphine (ppm) LC_50_ (95% confidence intervals) | Slope±SE | X^2^ | R | RF^1^ |
| --- | --- | --- | --- | --- | --- | --- |
| Wild-type |  | 195 (115-276) | 2.46±0.20 | 9.75 | 0.98 |  |
|  | UV | 266 (228-312) | 1.96±0.13 | 4.84 | 0.98 | 1.4 |
|  | Gamma-rays | 346 (294-404)^**^ | 1.80±0.16 | 3.91 | 0.99 | 1.8 |
| Phosphine-resistant *(dld-1(wr4))* |  | 1291(1130-1476) | 2.12±0.14 | 7.35 | 0.97 |  |
|  | UV | 2607 (1950-3705)^**^ | 2.48±0.186 | 11.37 | 0.99 | 2 |
|  | Gamma-rays | 2518 (2098-3108)^**^ | 1.51±0.13 | 3.47 | 0.99 | 2 |

^1^Resistance factor to the unpretreated worms for each strain (LC_50_ for pretreated/LC_50_ unpretreated).

**^**^*p* < 0.01.**
