# Supplementary material for: Stress pre-conditioning with temperature, UV and gamma radiation induces tolerance against phosphine toxicity
Source: PLoS One. 2018 Apr 19;13(4):e0195349. doi: 10.1371/journal.pone.0195349 (PMC5909616; doi:10.1371/journal.pone.0195349)
Supplement: S3 Table — (DOCX) [file pone.0195349.s004.docx]

S3 Table: UV induced mortality of wild-type and phosphine-resistant strains of *C. elegans*.

| Strain | UV (J cm^-2^) LD_50_ (95% confidence intervals) | Slope±SE | X^2^ | R | P value |
| --- | --- | --- | --- | --- | --- |

| Wild-type | 18 (12-23) | 2.5±0.23 | 12.95 | 0.96 | 0.01 |
| --- | --- | --- | --- | --- | --- |
| Phosphine-resistant *(dld-1(wr4))* | 33 (30-37) | 2.32±0.23 | 7.35 | 0.97 | 0.12 |
