# Supplementary material for: Stress pre-conditioning with temperature, UV and gamma radiation induces tolerance against phosphine toxicity
Source: PLoS One. 2018 Apr 19;13(4):e0195349. doi: 10.1371/journal.pone.0195349 (PMC5909616; doi:10.1371/journal.pone.0195349)
Supplement: S4 Table — A screening phosphine-bioassay in C. elegans mutants, that have been characterized in the C. elegans Genetic Center with genetic-mutated background in regard to heat shock response. Mutants with unique heat shock response to phosphine toxicity were chosen. (DOCX) [file pone.0195349.s005.docx]

**S4 Table: *C. elegans* mutants of the heat shock response were screened for a change in induced tolerance toward phosphine.** A screening phosphine-bioassay in *C. elegans* mutants, that have been characterized in the *C. elegans* Genetic Center with genetic-mutated background in regard to heat shock response. Mutants with unique heat shock response to phosphine toxicity were chosen

| Strain | Description | Genotype | Survival (%) | | | | | | | |
| --- | --- | --- | --- | --- | --- | --- | --- | --- | --- | --- |
|  |  |  | Normal | | | | Heat Shock | | | |
|  |  |  | Control | LC_10_ (80ppm) | LC_50_ (230ppm) | LC_90_ (1000ppm) | Control | LC_10_ (80ppm) | LC_50_ (230ppm) | LC_90_ (1000ppm) |
| VC281 | F38E11.2. Superficially wild type. Attribution: This strain was provided by the C. elegans Reverse Genetics Core Facility at the University of British Columbia, which is part of the international C. elegans Gene Knockout Consortium. | *hsp-12.6(gk156) IV* | 91 | 100 | 41 | 3 | 100 | 100 | 54 | 10 |
| PS3551 | Defects in egg laying. Do not grow at 25C. Do not distribute this strain; other labs should request it from the CGC. | *hsf-1(sy441) I* | 100 | 100 | 87 | 4 | 100 | 100 | 56 | 19 |
| RB791 | T27E4.3, T27E4.8. Homozygous. Outer Left Sequence: TGGCATTCCTTCCTTATTGC. Outer Right Sequence: TGAGAAGCCGAGTAGCTGGT. Inner Left Sequence: GTAAGGCTTTCTGCCGTTTG. Inner Right Sequence: TGAGGGCCCTGTAGAAGTTG. Inner primer WT PCR product: 3051. Attribution: This strain was provided by the C. elegans Gene Knockout Project at the Oklahoma Medical Research Foundation, which was part of the International C. elegans Gene Knockout Consortium. | *hsp-16.48(ok577) V* | 100 | 100 | 59 | 1 | 100 | 100 | 100 | 63 |
| RB1098 | F38E11.2 Homozygous. Outer Left Sequence: GTGACGATTCGAGAGCAACA. Outer Right Sequence: CGTGCGAAGATTGAACAGAA. Inner Left Sequence: TTCGAAGCTCAATGAACGAA. Inner Right Sequence: AGCCCAAGATGACAATGGAC. Inner Primer PCR Length: 2303. Estimated Deletion Size: about 700 bp. Attribution: This strain was provided by the C. elegans Gene Knockout Project at the Oklahoma Medical Research Foundation, which was part of the International C. elegans Gene Knockout Consortium. | *hsp-12.6(ok1077) IV* | 100 | 79 | 77 | 1 | 100 | 100 | 77 | 72 |
| RB1104 | C15H9.6 Homozygous. Outer Left Sequence: GGGGTAGGAGAGCCATTTTC. Outer Right Sequence: ACTTGGCCTTTTCCGATTTT. Inner Left Sequence: CGATCGTTTAGAGCTCGTCC. Inner Right Sequence: CCTGCCGTTTCCATAACAGT. Inner Primer PCR Length: 2947. Estimated Deletion Size: about 1300 bp. Attribution: This strain was provided by the C. elegans Gene Knockout Project at the Oklahoma Medical Research Foundation, which was part of the International C. elegans Gene Knockout Consortium. | *hsp-3(ok1083) X* | 76 | 100 | 54 | 52 | 100 | 99 | 66 | 56 |
| RB2600 | 22A3.2 Homozygous. Outer Left Sequence: ttgaaaatgtttcttcgggg. Outer Right Sequence: aattacaactgactcggcgg. Inner Left Sequence: tgccagaaacttccagttca. Inner Right Sequence: gccccttcagcataacgat. Inner Primer PCR Length: 1319. Estimated Deletion Size: about 400 bp. Attribution: This strain was provided by the C. elegans Gene Knockout Project at the Oklahoma Medical Research Foundation, which was part of the International C. elegans Gene Knockout Consortium. | *hsp-12.1(ok3622) I* | 100 | 100 | 82 | 0 | 100 | 100 | 60 | 22 |
| RB2612 | C14B9.1 Homozygous. Outer Left Sequence: tttcaggtccacaacaccaa. Outer Right Sequence: aaaatcatccctcgatgtgc. Inner Left Sequence: agttcgaggtcggacttgac. Inner Right Sequence: cattattcgtgcgttgatgc. Inner Primer PCR Length: 1096. Estimated Deletion Size: about 400 bp. Attribution: This strain was provided by the C. elegans Gene Knockout Project at the Oklahoma Medical Research Foundation, which was part of the International C. elegans Gene Knockout Consortium. | *hsp-12.2(ok3638) III* | 100 | 100 | 94 | 9 | 100 | 100 | 100 | 27 |
